# Prevalence of female sexual dysfunction in allied health workers: a cross-sectional pilot study in a tertiary hospital in Singapore

**DOI:** 10.1186/s12905-019-0829-8

**Published:** 2019-11-14

**Authors:** Farah Safdar, Chui Lee Julia Eng, Khin Lay Wai, Wan Shi Tey, Seng Bin Ang

**Affiliations:** Singapore, Singapore

## Abstract

**Background:**

Female sexual dysfunction (FSD) is increasingly being identified as a problem around the world. Women can have problems in various parts of the sexual cycle - desire, arousal, lubrication, orgasm or they may experience pain related to sexual activity. The only study involving Singapore with regard to sexual dysfunction in women, the Asian Global Studies of Sexual Attitudes and Behaviours in 2002, reported that Singapore had one of the lowest age-standardised sexual dysfunction rates of 32% compared with other Asian countries. This pilot study aims to evaluate the prevalence of female sexual dysfunction and to investigate the independent significant risk factors among allied health workers in a tertiary hospital in Singapore.

**Methods:**

A cross-sectional study where an anonymous questionnaire which included 19 questions in the FSFI (Female Sexual Function Index) was distributed to all allied health workers in a tertiary hospital in Singapore aged between 18 to 70 years old.

**Results:**

Three hundred thirty completed questionnaires were involved in analysis. 56.0% of women were found to have sexual dysfunction. A significant difference was found in the prevalence of FSD when comparing nurses to other allied health staff, where nurses had a decreased risk of developing FSD. Age was not found to be a significant risk factor in our study. Respondents below 40 years of age had significantly lower satisfaction scores than those above 40. Indians and Filipinos were found to have lower scores than the Chinese and Malay respondents in the lubrication (*p* = 0.02) and pain domains (*p* = 0.02).

**Conclusion:**

A significant proportion our female allied health workers suffer from sexual dysfunction. In this study, we found that the overall prevalence was independent of age, race and marital status. Nurses had a lower risk of developing FSD. We will need further studies to assess the prevalence of female sexual dysfunction in the general population, to evaluate the independent significant risk factors for developing FSD, in addition to classical risk factors, as well as to assess the psychological impact of this condition and whether people would be willing to seek help for such problems.

## Background

Female sexual dysfunction (FSD) is increasingly being identified as a problem around the world. Women can have problems in various parts of the sexual cycle - desire, arousal, lubrication, orgasm or they may experience pain related to sexual activity. These abnormalities collectively may be enough to interfere significantly with sexual function, resulting in dysfunction. Research suggests that FSD is multifactorial, with potential causes and risk factors such as age, educational level, community and religious values, relationship issues, obesity, psychological and psychiatric problems, medical conditions, gynaecological disorders, and medications [1, 2].

The only study involving Singapore with regard to sexual dysfunction in women, the Asian Global Studies of Sexual Attitudes and Behaviours in 2002, found that Singapore had the lowest rate of reported age-standardised sexual dysfunction of 32% amongst middle-aged and elderly women when compared with other Asian countries [3]. Since then, prevalence rates of FSD in Singapore have not been investigated or analysed and the impact on society remains unknown. The U.S. Food and Drug Administration (FDA) in 2013 interviewed 80 women with FSD on the impact of this condition on their daily life. They cited negative impacts on relationships, self-esteem, identity and mood. All of these affect quality of life and may even have consequences to society and nationally (possibly affecting birth rates, happiness index). More studies would be needed to establish correlation, but suffice to say that with a high prevalence of FSD, there could be a detrimental impact that extends beyond the individual.

Other Asian studies investigating FSD have demonstrated a varying range of prevalence rates, from 5.5% among healthcare workers in Malaysia [4] to 46.1% of women in Korea, of which 21.5% was self-reported as recognised [5]. Viswanathan et al. [6] and Singh et al. [7] have found that up to two thirds of the population in India suffers from sexual dysfunction, with barely a tenth of them identifying it as a problem. While the lack of consistent methodology and variability of the criteria applied for FSD definition partly explain why the prevalence rates differ in various studies, consensus is that there is a large community in Asia plagued by female sexual dysfunction [8].

The main aim of this study is to assess the prevalence of female sexual dysfunction among allied health workers in a tertiary hospital in Singapore and to determine the significant risk factors for FSD.

## Methods

A cross sectional study was conducted in a 830-bedded Tertiary Hospital in Singapore between May 2015 and August 2015. An anonymous, self-administered questionnaire was distributed to a total of approximately 1400 allied health workers, from the Departments of Nursing, Rehabilitation, Nutrition and Dietetics, Medical Social Services and Pharmacy. Participation was entirely voluntary and responses were obtained via central collection boxes to ensure anonymity. Our targeted sample population was based on all the allied health workers who were working in the hospital during the study period. The responses were collected only after formal working hours. This was particularly important as sex still remains a taboo topic in some parts of Asia.

The questionnaire contained basic demographic questions – age, marital status, race, vocation, frequency of sexual activity, as well as the 19 questions of the Female Sexual Function Index (FSFI) [9]. A sample of our questionnaire can be found in the supplementary files section (Additional file 1). This is a useful, validated tool in the assessment of sexual function that incorporates 6 domains – desire, arousal, lubrication, orgasm, satisfaction and pain. There are 2 to 4 questions per domain assessed as part of the FSFI. For the domain of desire, the 2 questions asked were about the frequency of feeling sexual desire and a rating of the level of sexual desire. For the domain of arousal, the 4 questions asked related to frequency, level of sexual arousal, confidence in becoming sexually aroused and how often they were satisfied with arousal during sexual activity. For the domain of lubrication, the 4 questions asked were on frequency, difficulty level to become lubricated, how often they could maintain the lubrication until completion of sexual intercourse and how difficult it was to maintain this lubrication. For the domain of orgasm, the 2 questions asked were related to frequency of attaining orgasm during sexual intercourse, and how difficult it was to reach an orgasm. For the domain of satisfaction, the 4 questions asked were about satisfaction levels with ability to orgasm, satisfaction levels with emotional closeness with partner, satisfaction levels with their sexual relationship and satisfaction levels with their overall sex life. For the last domain of pain, the 3 questions asked were related to frequency of pain felt during vaginal penetration, frequency of pain felt after vaginal penetration and the level of pain or discomfort felt during or after vaginal penetration. One option is selected per question, which gives an idea of the frequency with which the problem exists (almost always, most times, sometimes, a few times, almost never) or the severity (extremely difficult, very difficult, difficult, slightly difficult, not difficult) of the domain assessed. Satisfaction levels were also assessed with similar gradation (very satisfied, moderately satisfied, equally satisfied and dissatisfied, moderately dissatisfied, very dissatisfied). Respondents can also answer with the option “no sexual activity” for the questions. The answers are then weighted (multiplied by a corresponding factor) depending on the domain in question and calculated into an overall score. The minimum score is 2 and the maximum score attainable is 36.

The inclusion criterion was specified as any female allied healthcare worker between the ages of 18 and 70 who have a partner they are sexually active with. We excluded males, women without a partner and women who have never been sexually active. This was stated clearly in the participation information sheet as well as at the heading of the questionnaire. Incomplete questionnaires in which any of the 19 questions of the FSFI were not answered were excluded from analysis.

A clinical cut off score of 26.55 in accordance with the use of the FSFI was used to identify female sexual dysfunction in our study, this value was found to have good discriminant validity [10]. While many of the studies in various Asian countries employing the FSFI have used different cut-off scores, we employed the official, universal cut-off score for this pilot study.

### Statistical analysis

All statistical analyses were carried out using STATA (release 15.0; StataCorp, College Station, TX) statistical software. For descriptive analysis, categorical variables will be presented as numbers and percentages with 95% confidence interval (CI). Continuous variables will be presented as mean ± standard deviation (SD). The outcomes measured were reported as prevalence of FSD. Comparisons of specific FSD rates were made using statistical tests on the equality of proportions.

To investigate the independent significant risk factors of FSD, univariate and multivariate logistic regression (stepwise backward variable selection procedure) with robust variance estimator was applied.

The initial variable selection stage was performed using univariate logistic regression with robust sandwich variance estimator before proceeding to the multivariate logistic regression. In the variable selection stage, *P* ≤ 0.1 with odds ratio (OR) that excludes 1 will be used as a cutoff for statistical significance in order not to miss any potentially important predictors. Statistical significance remains the conventionally defined *p* ≤ 0.05 and OR that excludes 1 in the multivariate models.

To choose among competing models, the preferred final fitted logistic regression model will be selected, based on the likelihood ratio (LR) test. We also performed Hosmer-Lemeshow goodness of fit test for the model fitness; and we use the *P* > 0.05 in this goodness of fit test*.* We have also adjusted for all other clinically significant covariates in the final fitted model such as age, marital status, ethnicity, job description and presence of chronic medical conditions. The final estimated effect sizes were expressed as odds ratio (OR) with 95% Confidence Intervals (CI).

## Results

**Fig. 1:**
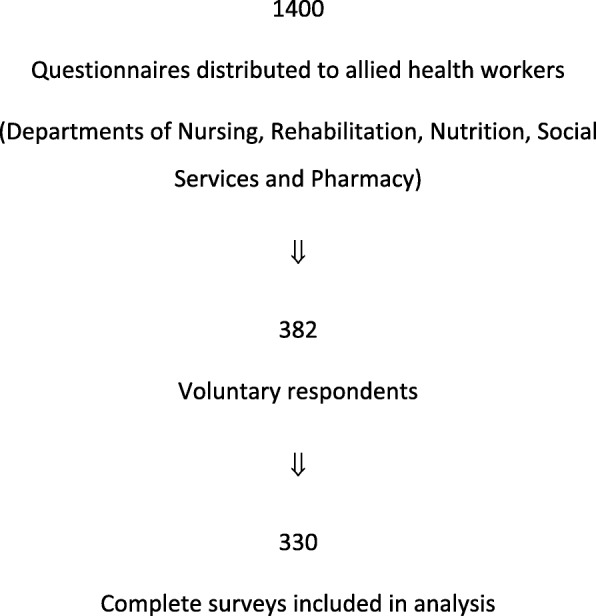
Flow diagram of participant response

Results were analysed from 330 respondents. See Fig. 1.

The following were the characteristics of the participants in our study.

- 303 (92%) participants were age 50 or less
- 117 (35%) participants were Chinese, 67 (21%) were Malay, 58 (18%) were Indian, 83 (26%) were classified as Others, of which 68 (21%) were Filipino
- 285 (86%) participants were married, 43 (14%) were either single, in a stable relationship or divorced
- 250 (76%) participants were from the Department of Nursing,10 (3%) were from Department of Rehabilitation, 4 (1%) were from Management, 1 (0%) was from Pharmacy, 1 (0%) was from Nutrition and Dietetics, and 60 (18%) were from other departments.
- 269 (82%) participants had no medical problems

In our study, we found that 185 of the 330 (56.0, 95% CI: 50.67–61.32%) respondents were classified as having female sexual dysfunction, with a FSFI score of < 26.55.

The mean scores across the different domains of sexual function are shown in Table 1.

**Table 1:** Mean scores on FSFI

Domain	Mean ± Standard Deviation
Desire	3.16 ± 1.11
Arousal	3.23 ± 1.87
Lubrication	3.68 ± 2.16
Orgasm	3.55 ± 2.17
Satisfaction	4.45 ± 1.51
Pain	3.76 ± 1.93
Total score	23.10 ± 8.80

Analysis was conducted across the independent variables to see if there was a significant difference between the patients classified as having female sexual dysfunction and those without (see Table 2). No significant difference was found for age, race, marital status and for the presence of chronic medical conditions. However, there was a significant difference in the prevalence of FSD between the nursing (52.4%) and other allied health staff (67.1%) groups.

**Table 2:** Relationship between variables and participants classified as with or without FSD

Variables	Normal (*n* = 145)		Female Sexual Dysfunction (*n* = 185)		
	N	%	N	%	*P*-value
Age					
0 to 40	113	77.93	134	72.83	0.336
41 to 50	24	16.55	32	17.39	
51 to 70	8	5.52	18	9.78	
Marital Status					
Married	127	87.59	158	86.34	0.74
Not Married	18	12.41	25	13.66	
Ethnicity					
Chinese	51	35.92	66	36.07	0.964
Malay	29	20.42	38	20.77	
Indian	24	16.9	34	18.58	
Other	38	26.76	45	24.59	
Job Description					
Nursing	119	82.64	131	71.98	**0.024**
Other allied health staff	25	17.36	51	28.02	
Chronic Medical Condition					
Yes	23	16.31	27	15.17	0.780
No	118	83.69	151	84.83	

The statistical significance threshold was set at *p* <0.05

Among the married subjects, we also found a statistically significant difference (*p* = 0.005), between nursing (50.9%) and the other allied health staff (70.8%) where the proportion of married nurses affected by sexual dysfunction was less when compared to other married allied health staff. Among non-married subjects, no difference was found between nurses and other allied health staff.

The univariate and multivariate logistic regression analysis showed that the nursing group had a significantly lower risk of FSD (adjusted OR 0.5, 95% CI 0.28–0.90, *p* = 0.020) when compared to the other allied health staff (reference group). See Table 3.

**Table 3:** Independent significant risk factors for FSD using univariate and multivariate logistic regression

Risk Factors	Univariate Analysis			Multivariate Analysis		
	Unadjusted Odds Ratio	95% CI	*p*	Adjusted Odds Ratio	95% CI	*p*
Age group						
< 51 years (Reference)	1			1		
> 51 years	1.34	0.72–2.50	0.349	1.23	0.60–2.50	0.577
Ethnicity						
Chinese (Reference)	1			1		
Malay	1.01	0.55–1.86	0.968	0.87	0.47–1.64	0.684
Indian	1.09	0.57–2.07	0.781	1.14	0.58–2.23	0.707
Others	0.91	0.52–1.61	0.758	0.95	0.53–1.73	0.877
Marital status						
Not married (Reference)	1			1		
Married	0.9	0.47–1.71	0.74	0.99	0.50–1.99	0.992
Job Description						
Other allied health staff (Reference)	1			1		
Nursing	0.54	0.31–0.93	**0.025**	0.5	0.28–0.90	**0.020**
Presence of Chronic Medical Conditions						
No (Reference)	1			1		
Yes	0.92	0.50–1.68	0.78	0.84	0.43–1.63	0.607

The statistical significance threshold was set at *p* <0.05

Among the patients with FSD, we looked into the various domains of female sexual function against the variables (see Table 4). Statistically significant differences were found between age and satisfaction score, where respondents below 40 years of age had lower satisfaction scores (mean scores + SD: age 21–30: 3.4 ± 1.5, age 31–40: 3.5 ± 1.4) than those above the age of 40 (mean scores: age 41–50: 4.0 ± 1.2, age 51 to 60 3.9 ± 1.0, age > 60 3.7 ± 0.5). We found significant differences (*p* = 0.018, *p* = 0.021) among our racial groups in the domains of pain and lubrication. The mean scores in the pain domain for Indians (2.6, ± 2.2) and Filipinos (2.5, ± 2.1) were lower than the Chinese (3.4 ± 1.9) and Malays (3.8 ± 1.7), and the mean scores for lubrication in Indians (2.4 ± 4.2) and Filipinos (2.4 ± 2.2) were also found to be lower than in the Chinese (3.3 ± 1.9) and Malays (3.4 ± 1.5).

**Table 4:** Demographic variables and the domains of sexual function

	*p*-value						
	Desire	Arousal	Lubrication	Orgasm	Satisfaction	Pain	Total Score
Age	0.1105	0.2836	0.5433	0.3224	**0.0228**	0.3158	0.2917
Race	0.8109	0.0934	**0.0182**	0.5756	0.587	**0.0212**	0.1393
Marital Status	0.4304	0.2959	0.5624	0.2069	0.3496	0.4038	0.4731
Job description	0.382	0.1287	0.4469	0.5355	0.2217	0.0815	0.1556

The statistical significance threshold was set at *p* <0.05

## Discussion

### Main findings

Our findings of a prevalence of 56.0% allied health workers suffering from female sexual dysfunction seemed to be higher than most other prevalence findings in Asia, which is a contrast to the study mentioned earlier, done in 2002 where Singapore was reported to have one of the lowest sexual dysfunction rates among all Asian countries studied [3].

We did not find any significant difference across the variables of age, marital status, race or the presence of any chronic medical condition. We did, however, find a significant difference between nursing staff and non-nursing staff, where nurses had a lower prevalence rate of FSD. More needs to be done to investigate this association – whether it is related to the nature of their work or schedules, awareness of the human body, or simply just a lack of disclosure.

Age has been identified as a risk factor for FSD with increasing age posing increased risk across most studies [11, 12]. However, age was not a statistically significant variable (*p* = 0.336) in assessing FSD in our study. There was no significant difference even with segregating the groups as pre-menopausal (age 50 and below) and post menopausal (age 51 and above). The mean age of menopause in among women in Singapore is 49, and we used this as a proxy for our estimations [13]. While most studies suggest that menopause and aging have an inverse relationship with sexual function, there have been a few that showed the opposite relationship [14] – citing lack of fear of pregnancy and being with their partners for longer as a possible reason. Our finding may possibly be accounted for by the small sample size of the respondents who were over 50 years old (*n* = 26).

Assessment of the variables as risk factors revealed that nurses were less likely to develop FSD than the other allied health staff. Both univariate and multivariate analysis revealed an odds ratio of approximately 0.5 (*p* = 0.020, *p* = 0.025). Age, marital status, race and the presence of chronic medical conditions did not appear to be risk factors for the development of FSD in our study.

An interesting finding on analysis of the individual domains of sexual function was the difference among our racial groups in the domains of pain and lubrication (*p* = 0.018, *p* = 0.021), with Indians and Filipinos having lower scores than Chinese and Malays. This could suggest that race has a part to play in certain domains of sexual function and different races are subject to different sets of problems with sexual activity. This could be attributed to physical and physiological differences, cultural differences or perhaps diet. This is in keeping with the discrepant prevalence rates of sexual dysfunction seen across Asia – where the Chinese population in Hong Kong and China have found rates of FSD between 25.6 to 37.9% [15, 16], Malaysian studies identified prevalence rates of between 5.5% [4] among healthcare workers and 29.6% in the primary care setting and Indian studies have seen up to 64% of the population as having dysfunction [6, 7].

We did, however, find that the women below the age of 40 years scored lower in the satisfaction domain than did the group above the age of 40 (*p* = 0.023). There has been some conflicting data on sexual satisfaction and its relationship to aging. Some studies have found that increasing age is associated with better sexual satisfaction [17], in spite of infrequent sexual activity [18], while others have failed to demonstrate a correlation with age [19]. This may be related to less expectation in sexual performance as a consequence of aging.

## Strengths and limitations

### Study design

This is the first study on female sexual dysfunction in allied health workers in Singapore. We conducted a cross-sectional study to understand the prevalence of FSD among our allied health workers and this study design was an efficient way of evaluating a large sample of workers. Our selection of intended participants, within one hospital setting, and the need for convenience would have induced a sampling bias, which may have resulted in an artificially larger number of patients classified as having FSD. Other studies done in Asia which had a more general sample population have found quite varied prevalence rates ranging from 25.6% [15] in Hong Kong to 64.3% [6] in India. Our results were found to be within this range, despite the sampling bias.

We had a 25% response rate, which is slightly above the expected of around 20% [20] for such a study design. However, our sample size could have been larger in order to ensure that the demographic profile of the survey respondents reflected that of the survey population to reduce selection bias and to provide a larger data set for analysis.

There were potential confounders to this study that were not addressed or corrected for, for example – smoking and alcohol history, partner factors, mood and stability of the relationship, body mass index.

While the cross-sectional study was useful in providing an understanding of the prevalence rates of FSD among allied health workers, it also had its limitations. As with all other cross-sectional studies, temporal relationships between the demographic variables and the development of FSD cannot be established and therefore causality is unclear [21].

## Questionnaire

We only accepted questionnaires in which all the 19 questions of the FSFI were filled, our primary aim of determining the prevalence of FSD among allied health workers in a tertiary hospital was achieved. However, in order to maintain a sizeable sample, we also included questionnaires in the study even if the demographic questions were not fully filled. This could have potentially skewed the analysis of the data for our secondary aim, which was to analyse if demographic variables were associated with the prevalence rates of FSD among the respondents. Also, we did not obtain a full medical background or ask about the presence of pregnancy for the respondents, which could be contributory to their sexual function.

### Data analysis

The FSFI cut off score of 26.55 was recommended under the circumstance of a different population. The Asian population may not conform to that cut off score, even if it did show good discriminant validity – hence, there could either be an underestimation or overestimation.

The large proportion of women who did not have any sexual activity in the preceding month although they claimed to be sexually active may have led to false positives due to the low values obtained in the FSFI. We also did not take into account misinterpretation of penetrative sexual intercourse as implied by the FSFI to other kinds of sexual activity.

## Interpretation

The prevalence of FSD in the allied health population cannot be extrapolated to the general population as it is not a representative sample in view of inherent differences, but it does serve as a useful platform for further studies to be done. While previous studies have shown that FSD is more prevalent among working women, healthcare workers were at particular risk due to their working environment and patterns of work schedules [8]. This could possibly account for the higher than average prevalence rate found in the study.

## Conclusion

There is a high prevalence (56%) of female sexual dysfunction in allied health workers between the ages of 21 to 70 in a tertiary hospital in Singapore, and this was independent of age, race, marital status and presence of chronic medical conditions. Nurses were found to have a lower prevalence rate than their other allied health counterparts, and were less likely to develop female sexual dysfunction.

Analysis across the domains of sexual dysfunction revealed that Indians and Filipinos tend to have less problems with lubrication and pain. It also found higher satisfaction with sex life for women above 40.

The diverse population in Singapore would make it a suitable place for more detailed assessment on the impact of race on female sexual function.

We will need further population studies with a wider sample size to assess the prevalence of female sexual dysfunction in the general population, to identify associated risk factors, assess the psychological impact of this condition and determine if people would be willing to seek help for such problems.

## Supplementary information

**Additional file 1.** FSD Questionnaire. Questionnaire used in study titled: Female Sexual Dysfunction in Singapore – How much of a burden is it?. The questionnaire attached contains basic demographic data, and includes the 19 questions in the Female Sexual Function Index.

## Data Availability

The datasets used and analysed during the current study are available from the first author on reasonable request.

## Abbreviations

FSD: Female Sexual Dysfunction
FSFI: Female Sexual Function Index
