# Supplementary material for: Prevalence of female sexual dysfunction in allied health workers: a cross-sectional pilot study in a tertiary hospital in Singapore
Source: BMC Womens Health. 2019 Nov 14;19:137. doi: 10.1186/s12905-019-0829-8 (PMC6854745; doi:10.1186/s12905-019-0829-8)
Supplement: Supplementary file 1 — Additional file 1. FSD Questionnaire. Questionnaire used in study titled: Female Sexual Dysfunction in Singapore – How much of a burden is it?. The questionnaire attached contains basic demographic data, and includes the 19 questions in the Female Sexual Function Index. [file 12905_2019_829_MOESM1_ESM.pdf]

Female Sexual Dysfunction in Singapore - How much of a burden is it?
A study of allied health workers in KK Women and Children's Hospital

This questionnaire is for women above 18 who have a partner they are sexually active with. Please discard the questionnaire if it is not applicable to you.

Age:

☐ 21 – 25	☐ 31 – 35	☐ 41 – 45	☐ 51 – 55	☐ 60 – 65
☐ 26 – 30	☐ 36 – 40	☐ 46 – 50	☐ 56 – 60	☐ 65 – 70

Race:

☐ Chinese	☐ Indian	☐ Filipino
☐ Malay	☐ Eurasian	☐ Others

Marital status:

☐ Single	☐ Married	☐ Others: ______________
☐ Divorced	☐ Stable relationship	

Gender of partner:

☐ Male
☐ Female

Job description:

☐ Management	☐ Nursing	☐ Pharmacy
☐ Nutrition and Dietetics	☐ Rehabilitation	☐ Others: ______________

Medical conditions:

☐ Hypertension	☐ Diabetes	☐ NIL
☐ Depression	☐ Hyperlipidemia	☐ Others: ______________

---

1. Frequency of sexual intercourse with partner:
   - ☐ Less than once a month
   - ☐ Once to twice a month
   - ☐ Once to twice a week
   - ☐ Three to four times a week
   - ☐ More than four times a week

Sexual desire or interest is a feeling that includes wanting to have a sexual experience, feeling receptive to a partner's sexual initiation, and thinking or fantasizing about having sex.

2. Over the past 4 weeks, how **often** did you feel sexual desire or interest?
   - ☐ Almost always or always
   - ☐ Most times (more than half the time)
   - ☐ Sometimes (about half the time)
   - ☐ A few times (less than half the time)
   - ☐ Almost never or never

3. Over the past 4 weeks, how would you rate your **level** (degree) of sexual desire or interest?
   - ☐ Very high
   - ☐ High
   - ☐ Moderate
   - ☐ Low
   - ☐ Very low or none at all

Sexual arousal is a feeling that includes both physical and mental aspects of sexual excitement. It may include feelings of warmth or tingling in the genitals, lubrication (wetness), or muscle contractions.

4. Over the past 4 weeks, how **often** did you feel sexually aroused ("turned on") during sexual activity or intercourse?
   - ☐ No sexual activity
   - ☐ Almost always or always
   - ☐ Most times (more than half the time)
   - ☐ Sometimes (about half the time)
   - ☐ A few times (less than half the time)
   - ☐ Almost never or never

5. Over the past 4 weeks, how would you rate your **level** of sexual arousal ("turn on") during sexual activity or intercourse?
   - ☐ No sexual activity
   - ☐ Very high
   - ☐ High
   - ☐ Moderate
   - ☐ Low
   - ☐ Very low or none at all

6. Over the past 4 weeks, how **confident** were you about becoming sexually aroused during sexual activity or intercourse?
   - ☐ No sexual activity
   - ☐ Very high confidence
   - ☐ High confidence
   - ☐ Moderate confidence
   - ☐ Low confidence
   - ☐ Very low or no confidence

7. Over the past 4 weeks, how **often** have you been satisfied with your arousal (excitement) during sexual activity or intercourse?
   - ☐ No sexual activity
   - ☐ Almost always or always
   - ☐ Most times (more than half the time)
   - ☐ Sometimes (about half the time)
   - ☐ A few times (less than half the time)
   - ☐ Almost never or never

8. Over the past 4 weeks, how **often** did you become lubricated ("wet") during sexual activity or intercourse?
   - ☐ No sexual activity
   - ☐ Almost always or always
   - ☐ Most times (more than half the time)
   - ☐ Sometimes (about half the time)
   - ☐ A few times (less than half the time)
   - ☐ Almost never or never

9. Over the past 4 weeks, how **difficult** was it to become lubricated ("wet") during sexual activity or intercourse?
   - ☐ No sexual activity
   - ☐ Extremely difficult or impossible
   - ☐ Very difficult
   - ☐ Difficult
   - ☐ Slightly difficult
   - ☐ Not difficult

10. Over the past 4weeks, how often did you **maintain** your lubrication ("wetness") until completion of sexual activity or intercourse?
- ☐ No sexual activity
- ☐ Almost always or always
- ☐ Most times (more than half the time)
- ☐ Sometimes (about half the time)
- ☐ A few times (less than half the time)
- ☐ Almost never or never

11. Over the past 4 weeks, how **difficult** was it to maintain your lubrication ("wetness") until completion of sexual activity or intercourse?
- ☐ No sexual activity
- ☐ Extremely difficult or impossible
- ☐ Very difficult
- ☐ Difficult
- ☐ Slightly difficult
- ☐ Not difficult

12. Over the past 4 weeks, when you had sexual stimulation or intercourse, how **often** did you reach orgasm (climax)?
- ☐ No sexual activity
- ☐ Almost always or always
- ☐ Most times (more than half the time)
- ☐ Sometimes (about half the time)
- ☐ A few times (less than half the time)
- ☐ Almost never or never

13. Over the past 4 weeks, when you had sexual stimulation or intercourse, how **difficult** was it for you to reach orgasm (climax)?
- ☐ No sexual activity
- ☐ Extremely difficult or impossible
- ☐ Very difficult
- ☐ Difficult
- ☐ Slightly difficult
- ☐ Not difficult

14. Over the past 4 weeks, how **satisfied** were you with your ability to reach orgasm (climax) during sexual activity or intercourse?
- ☐ No sexual activity
- ☐ Very satisfied
- ☐ Moderately satisfied
- ☐ About equally satisfied and dissatisfied
- ☐ Moderately dissatisfied
- ☐ Very dissatisfied

15. Over the past 4 weeks, how **satisfied** have you been with the amount of emotional closeness during sexual activity between you and your partner?
- ☐ No sexual activity
- ☐ Very satisfied
- ☐ Moderately satisfied
- ☐ About equally satisfied and dissatisfied
- ☐ Moderately dissatisfied
- ☐ Very dissatisfied

16. Over the past 4 weeks, how **satisfied** have you been with your sexual relationship with your partner?
    - ☐ Very satisfied
    - ☐ Moderately satisfied
    - ☐ About equally satisfied and dissatisfied
    - ☐ Moderately dissatisfied
    - ☐ Very dissatisfied

17. Over the past 4 weeks, how **satisfied** have you been with your overall sexual life?
    - ☐ Very satisfied
    - ☐ Moderately satisfied
    - ☐ About equally satisfied and dissatisfied
    - ☐ Moderately dissatisfied
    - ☐ Very dissatisfied

18. Over the past 4 weeks, how **often** did you experience discomfort or pain **during** vaginal penetration?
    - ☐ Did not attempt intercourse
    - ☐ Almost always or always
    - ☐ Most times (more than half the time)
    - ☐ Sometimes (about half the time)
    - ☐ A few times (less than half the time)
    - ☐ Almost never or never

19. Over the past 4 weeks, how **often** did you experience discomfort or pain **following** vaginal penetration?
    - ☐ Did not attempt intercourse
    - ☐ Almost always or always
    - ☐ Most times (more than half the time)
    - ☐ Sometimes (about half the time)
    - ☐ A few times (less than half the time)
    - ☐ Almost never or never

20. Over the past 4 weeks, how would you rate your **level** (degree) of discomfort or pain during or following vaginal penetration?
    - ☐ Did not attempt intercourse
    - ☐ Very high
    - ☐ High/Moderate
    - ☐ Low
    - ☐ Very low or none at all

Thank you for completing this questionnaire.
